# Infectious retinitis associated with JAK inhibitors: the ongoing duality of JAK inhibitors

**DOI:** 10.1186/s40942-026-00823-4

**Published:** 2026-03-06

**Authors:** Jose S. Pulido, Fukutaro Mano, Marcello Casella, Susumu Ishida

**Affiliations:** 1 Larry Donoso Chair of Translational Ophthalmology, Wills Eye Hospital, Thomas Jefferson University, Philadelphia, PA USA; 2 Departments of Ophthalmology and Molecular Medicine, Mayo Clinic, Rochester, USA; 3 Department of Ophthalmology, Kindai University Faculty of Medicine, Sakai, Osaka Japan; 4 Departament of Ophthalmology, Estadual de Londrina, UEL, Londrina, PR Brazil; 5 Department of Ophthalmology, Faculty of Medicine and Graduate School of Medicine, Hokkaido University, Sapporo, Japan

**Keywords:** Cytomegalovirus, Cytomegalovirus retinitis, Immunosuppression, Immunosuppressant therapy, Opportunistic infection, Rheumatoid arthritis, Tofacitinib, Valganciclovir, Viral retinitis, Xeljanz

## Abstract

**Purpose:**

To discuss the recent evidence that Janus kinase (JAK) inhibitors can cause viral retinitis.

**Methods:**

A commentary on the case report in this issue and a review of cases in the literature.

**Results:**

A total of 6 cases have now been reported of cytomegalovirus retinitis associated with Jak inhibitors. Additionally, there was a case of bilateral toxoplasma retinitis and a case of herpetic scleritis.

**Discussion:**

It is important to be aware of the potential blinding complications associated with JAK inhibitors and if the patient complains of acute vision loss and/or floaters, the patient should be emergently referred to a retina specialist.

## Background

In this issue Watanabe and coauthors describe a patient who developed progressive outer retinal necrosis from Herpes Zoster [1]. Recently, others have shown that viral herpesviridiae retinitis occurs usually in patients that have autoimmune disorders for which Janus kinase (JAK) inhibitors were used [2–6]. Both herpes and cytomegalovirus (CMV) retinitis have now been described though most cases have been CMV-associated [2]. Additionally there have been a case of bilateral toxoplasma retinitis [7] and another of herpetic scleritis [8]. 

## Main text

JAK inhibitors are becoming more frequently used as first-line therapy in a wide variety of autoimmune and malignant diseases. It is hard to determine the current use of JAK inhibitors, but it is estimated that in 2024, $ 20 billion was spent on JAK inhibitors in the USA (Janus Kinase (JAK) Inhibitors Market 2025; Overview and Share) [9]. They are actually being used in patients with noninfectious uveitis, and a randomized controlled trial is currently underway for this indication [10]. Because of the widespread use of these agents, it is important for ophthalmologists to understand how they are used and their complications.

The JAK/STAT pathway is activated by multiple receptors. These receptors are activated by hormones, growth factors and cytokines. Once the receptor is activated, the JAK protein which is bound to the receptor then phosphorylates the Stat proteins. The phosphorylated STAT proteins then interact with the DNA to cause transcription of a multitude of transcripts including pro-inflammatory agents. Agents that inhibit JAK prevent the downstream activation of this pathway.

Inhibition is approved for use in a variety of disorders. These can be divided into JAK-associated hematopoietic disorders, graft-versus-host disease, and autoimmune diseases. The hematopoietic disorders are associated with JAK mutations. These include myelofibrosis and polycythemia vera. Autoimmune diseases for which JAK inhibitors are approved include: rheumatoid arthritis, psoriatic arthritis, juvenile idiopathic arthritis, ulcerative colitis, Crohn’s disease, ankylosing spondylitis, and giant cell arteritis. The last of these indications is important for recognition by ophthalmologists since there are few other medications that are approved for giant cell arteritis, namely tocilizumab and, of course, corticosteroids.

All JAK inhibitors have a black box warning about severe adverse events that include: major adverse cardiovascular events (MACE) [11], higher all-cause mortality, including sudden cardiovascular death, thrombosis, malignancies, and infections [12]. The types of systemic infections include bacterial (including tuberculosis), fungal, and viral. Progressive multifocal leukoencephalopathy, caused by the JC polyoma virus, is a rare but well-known complication. More common are systemic herpesvirus infections especially herpes simplex and herpes zoster. Summarising the retinal complications of 8 published cases, the age of onset ranges from 48 to 82. Older ages were mostly affected, but the youngest one was 48 years old. More than half (5 cases) were treated solely with JAK inhibitors, and others were simultaneously treated with prednisolone (*n* = 2) and methotrexate (*n* = 3). The type of JAK inhibitor also varies; tofacitinib (*n* = 4), ruxolitinib (*n* = 3), and upadacitinib (*n* = 1). These results suggest the specific JAK inhibitor may not be responsible for the ocular complications (Table 1).

**Table 1:** Summary of patient profile and retinal complications related to possible JAK inhibitors

Authors and published year	Age	Original disease	Retinal complications	Specific JAK inhibitor	Concurrent immunosuppression
Watanabe et al. 2025	48	pulmonary graft-versus-host disease	VZV-associated PORN	ruxolitinib	prednisolone (11 mg/day)
Smith et al. 2025	79	rheumatoid arthritis	CMV retinitis	tofacitinib	None
Smith et al. 2025	82	rheumatoid arthritis	CMV retinitis	tofacitinib	None
Cugley et al. 2020	54	rheumatoid arthritis	CMV retinitis	tofacitinib	methotrexate (15 mg/week), prednisolone (15 mg/day)
Hirai et al. 2023	79	rheumatoid arthritis	CMV retinitis	upadacitinib	None
Yanagisawa et al. 2017	78	rheumatoid arthritis	CMV retinitis	tofacitinib	Methotrexate (dose unknown)
von Hofsten et al. 2016	67	primary myelofibrosis	CMV retinitis	ruxolitinib	None
Goldberg et al. 2013	65	primary myelofibrosis	toxoplasmosis retinitis	ruxolitinib	None

CMV; Cytomegalovirus, JAK; Janus kinase, PORN; Progressive outer retinal necrosis, VZV; Varicella zoster virus

Because there are now cases of CMV retinitis [2–6], toxoplasma retinitis [7], and now herpes retinitis [1, 8], it is important to educate not only our fellow ophthalmologists but also our medical colleagues who prescribe these medications that these patients should be immediately referred to a retina specialist if they notice acute vision loss and/or floaters. Janus was the Roman god that looked backwards and forwards. He had two faces, and now these JAK inhibitors have the same duality. JAK inhibitors hold great promise, but they also have the possibility of great harm as well. Both faces need to be considered.

## Data Availability

Not applicable.

## Abbreviations

CMV: Cytomegalovirus
DNA: Deoxyribonucleic Acid
JAK: Janus kinase
MACE: Major adverse cardiovascular events

## References

[1] Watanabe D, Namba K, Hase K, Suzuki K, Ogino Y, Saito M, et al. Acute retinal necrosis during the systemic use of Janus kinase inhibitor. Int J Retina Vitreous. 2026. 10.1186/s40942-025-00756-4 PMC12679767 41345747

[2] Smith MJ, Lim JI, Chau FY, Lobo-Chan AM. Cytomegalovirus Retinitis in Non-HIV Immunocompromised Individuals on Tofacitinib for Rheumatoid Arthritis. Retinal Cases Brief Rep. 2025;19(5):603–7. 10.1097/ICB.0000000000001626 PMC12961194 41779881

[3] Cugley DR, Darby J, Lim LL. Tofacitinib-associated cytomegalovirus retinitis. Rheumatology (Oxford). 2020;59:e35–7. 31990341 10.1093/rheumatology/kez681

[4] Hirai H, Akai Y, Ogata N, Ueda T. Cytomegalovirus Retinitis in a Patient Taking Upadacitinib: A Case Report. Cureus. 2023;15:e48337. 38060716 10.7759/cureus.48337 PMC10698290

[5] Yanagisawa K, Ogawa Y, Hosogai M, Todokoro D, Mitsui T, Yokohama A, et al. Cytomegalovirus retinitis followed by immune recovery uveitis in an elderly patient with rheumatoid arthritis undergoing administration of methotrexate and tofacitinib combination therapy. J Infect Chemother. 2017;23:572–5. 28389165 10.1016/j.jiac.2017.03.002

[6] von Hofsten J, Johnsson Forsberg M, Zetterberg M. Cytomegalovirus Retinitis in a Patient Who Received Ruxolitinib. N Engl J Med. 2016;374:296–7. 10.1056/NEJMc1413918 26789901

[7] Goldberg RA, Reichel E, Oshry LJ. Bilateral toxoplasmosis retinitis associated with ruxolitinib. N Engl J Med. 2013;369:681–3. 23944322 10.1056/NEJMc1302895

[8] Pyare R, Dutta MP, Shah M, Kaushik V, Agarwal M, Biswas J. Tofacitinib in Scleritis: A Case Series. Ocul Immunol Inflamm. 2024;32:884–90. 36126052 10.1080/09273948.2022.2113805

[9] The Janus Kinase Inhibitor Global Market Report. 2025. https://www.thebusinessresearchcompany.com/report/janus-kinase-jak-inhibitors-global-market-report. Accessed 29 Nov 2025.

[10] Srivastava SK, Watkins TR, Nguyen QD, Sharma S, Scales DK, Dacey MS, et al. Filgotinib in Active Noninfectious Uveitis: The HUMBOLDT Randomized Clinical Trial. JAMA Ophthalmol. 2024;142:789–97. 39023880 10.1001/jamaophthalmol.2024.2439 PMC11258638

[11] Di Napoli R, Richez C, Scavone C, Singier A, Demourgues M, Mascolo A, et al. Major Adverse Cardiovascular Events Related to JAK Inhibitors: A Disproportionality Analysis Using the WHO Global Individual Case Safety Database. Drug Saf. 2025;48:943–52. 40121611 10.1007/s40264-025-01535-8 PMC12259736

[12] Hoisnard L, Lebrun-Vignes B, Maury S, Mahevas M, El Karoui K, Roy L, et al. Adverse events associated with JAK inhibitors in 126,815 reports from the WHO pharmacovigilance database. Sci Rep. 2022;12:7140. 35504889 10.1038/s41598-022-10777-w PMC9065106

